# Effect of Mailed Human Papillomavirus Test Kits vs Usual Care Reminders on Cervical Cancer Screening Uptake, Precancer Detection, and Treatment: A Randomized Clinical Trial

**DOI:** 10.1001/jamanetworkopen.2019.14729

**Published:** 2019-11-06

**Authors:** Rachel L. Winer, John Lin, Jasmin A. Tiro, Diana L. Miglioretti, Tara Beatty, Hongyuan Gao, Kilian Kimbel, Chris Thayer, Diana S. M. Buist

**Affiliations:** 1 Department of Epidemiology, University of Washington, Seattle; 2 Kaiser Permanente Washington Health Research Institute, Seattle; 3 Department of Clinical Sciences, University of Texas Southwestern Medical Center, Dallas; 4 Division of Biostatistics, Department of Public Health Sciences, University of California, Davis; 5 Kaiser Permanente Washington, Renton

## Abstract

**Importance:**

In the United States, more than 50% of cervical cancers are diagnosed in underscreened women. Cervical cancer screening guidelines now include primary human papillomavirus (HPV) testing as a recommended strategy. Home-based HPV self-sampling is a viable option for increasing screening compliance and effectiveness; however, US data are needed to inform health care system implementation.

**Objective:**

To evaluate effectiveness of mailed HPV self-sampling kits vs usual care reminders for in-clinic screening to increase detection and treatment of cervical intraepithelial neoplasia grade 2 or worse (CIN2+) and uptake of cervical cancer screening.

**Design, Setting, and Participants:**

Randomized clinical trial conducted in Kaiser Permanente Washington, a US integrated health care delivery system. Women aged 30 to 64 years with health plan enrollment for 3 years and 5 months or more, a primary care clinician, no Papanicolaou test within 3 years and 5 months, and no hysterectomy were identified through electronic medical records and enrolled from February 25, 2014, to August 29, 2016, with follow-up through February 26, 2018.

**Interventions:**

The control group received usual care (annual patient reminders and ad hoc outreach from primary care clinics). The intervention group received usual care plus a mailed HPV self-sampling kit.

**Main Outcomes and Measures:**

Two primary outcomes were (1) CIN2+ detection within 6 months of screening and (2) treatment within 6 months of CIN2+ detection. Screening uptake within 6 months of randomization was a secondary outcome.

**Results:**

A total of 19 851 women (mean [SD] age, 50.1 [9.5] years) were included, with 9960 randomized to the intervention group and 9891 randomized to the control group. All women randomized were included in analysis. In the intervention group, 12 participants with CIN2+ were detected compared with 8 in the control group (relative risk, 1.49; 95% CI, 0.61-3.64) and 12 cases were treated vs 7 in the control group (relative risk, 1.70; 95% CI, 0.67-4.32). Screening uptake was higher in the intervention group (2618 participants [26.3%] vs 1719 participants [17.4%]; relative risk, 1.51; 95% CI, 1.43-1.60).

**Conclusions and Relevance:**

Mailing HPV kits to underscreened women increased screening uptake compared with usual care alone, with no significant differences in precancer detection or treatment. Results support the feasibility of mailing HPV kits to women who are overdue for screening as an outreach strategy to increase screening uptake in US health care systems. Efforts to increase kit uptake and follow-up of positive results are warranted to maximize detection and treatment of CIN2+.

**Trial Registration:**

ClinicalTrials.gov identifier: NCT02005510

## Key Points

**Question:**

Do mailed human papillomavirus self-sampling kits increase detection and treatment of cervical precancers and screening uptake vs usual care (reminders for in-clinic screening)?

**Findings:**

This randomized clinical trial included 19 851 women; 26% were screened after receiving a human papillomavirus kit vs 17% with usual care, a significant difference. There was no statistically significant difference in the number of cases of precancers detected or treated.

**Meaning:**

This study indicates that mailing human papillomavirus kits to underscreened women can increase cervical cancer screening, and implementation efforts should strategize how to further increase kit uptake and follow-up of positive results to maximize detection and treatment of precancers in women at high risk.

## Teaser

This randomized clinical trial evaluates the effectiveness of mailed self-test kits for human papillomavirus (HPV) vs usual care for detection and treatment of cervical precancers and uptake of cervical cancer screening.

## Introduction

In the United States, 25% of women delay or forego recommended cervical cancer screening.^1,2,3^ Well-documented barriers include lack of time or transportation, difficulties finding childcare or taking time off work, fear of pelvic examinations, and prior negative experiences with screening.^4,5,6,7,8,9^ Reducing underscreening is a key prevention priority,^10^ as more than 50% of the 12 000 cervical cancers diagnosed annually are in underscreened women.^9,11,12,13^

In 2018, the US Preventive Services Task Force released updated cervical cancer screening guidelines^14^ that include 3 recommended options for women aged 30 to 65 years: Papanicolaou testing alone, Papanicolaou and human papillomavirus (HPV) cotesting, and primary HPV-only screening (a new strategy). With primary HPV screening, home-based screening is an emerging option because HPV tests (unlike Papanicolaou tests) can be performed on clinician- or self-collected samples with comparable sensitivity.^15,16^ Furthermore, population-based randomized clinical trials in countries with organized screening programs (ie, centrally designed and managed, with standardized screening invitations and follow-up for a specific target population) demonstrated that mailing HPV self-sampling kits to underscreened women increased participation compared with invitations for clinic-based screening^15^ and diagnostic follow-up compliance after HPV-positive self-sampling results was high,^15^ yielding increased detection of cervical intraepithelial neoplasia grade 2 or higher (CIN2+).^15^ Consequently, Australia and the Netherlands—the first countries to implement primary HPV screening—have included HPV self-sampling options for underscreened women.^17,18^

Data from settings without organized screening programs—including opportunistic and/or health system–based screening programs in the United States—are needed to evaluate whether home-based HPV self-sampling increases screening participation and effectiveness. The Home-Based Options to Make Cervical Cancer Screening Easy (HOME) trial^19^ compared a programmatic strategy of mailed HPV self-sampling kits with usual care (outreach via patient reminders to attend in-clinic screening) for increasing detection and treatment of cervical precancers and uptake of cervical cancer screening. The HOME study is the first US trial, to our knowledge, to evaluate effectiveness of HPV self-sampling as a screening method by assessing the entire cervical cancer prevention process (diagnostic follow-up and treatment) and measuring precancer outcomes. To our knowledge, it is also the first HPV self-sampling trial in any setting to evaluate treated precancer cases as an outcome.

## Methods

**Figure 1.**
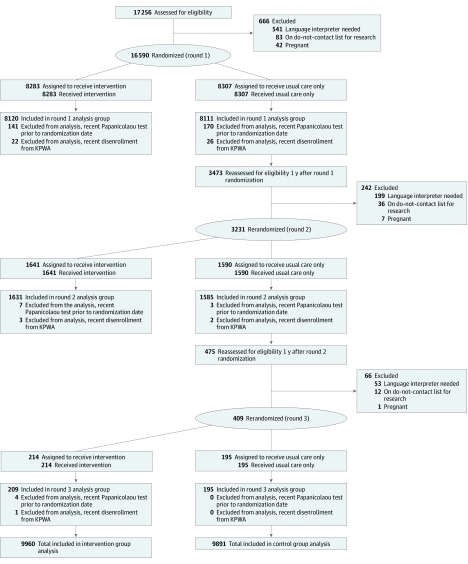
CONSORT Diagram We identified 63 789 women who were between the ages of 30 and 64 years, had not had a hysterectomy, had a primary care clinician at Kaiser Permanente Washington (KPWA), and had been continuously enrolled in the health system for at least 3 years and 5 months. Of these women, 17 256 (27.1%) had not had a Papanicolaou test within 3 years and 5 months. Postrandomization exclusions were applied owing to delays in data warehouse update. The total included in the intervention group analysis (9960) equals the total included in the round 1 analysis group (8120) plus the total included in the round 2 analysis group (1631) plus the total included in the round 3 analysis group (209). Similarly, the total included in the control group analysis (9891) equals the total included in the round 1 analysis group (8111) plus the total included in the round 2 analysis group (1585) plus the total included in the round 3 analysis group (195).

The HOME study was a parallel, investigator-blinded, randomized clinical trial comparing 2 programmatic strategies for improving screening effectiveness and uptake in women who were not adherent to routine Papanicolaou screening. The trial was fully embedded within Kaiser Permanente Washington (KPWA) (see the article by Winer et al^19^ for details about pragmatic aspects according to Pragmatic-Explanatory Continuum Indicator Summary–2 [PRECIS-2] criteria^20^) and was approved by KPWA and University of Washington institutional review boards. The trial followed the Consolidated Standards of Reporting Trials (CONSORT) reporting guideline (Figure 1). The trial protocol is available in Supplement 1.

We identified potentially eligible women using electronic medical record (EMR) data; all eligible women were enrolled under a waiver of informed consent. No incentives were offered for participation. The institutional review boards determined that enrolling women under a waiver of informed consent was minimal risk compared with the scientific benefit of reducing participation bias. Women became eligible 5 months after receiving an annual preventive care reminder letter indicating they were due or overdue for their Papanicolaou screening (ie, no prior screening or most recent screening >3 years prior) to allow women to achieve screening uptake without additional intervention and to ensure that the 6-month time frame for assessing screening uptake ended before the next year’s preventive care reminder letter was sent. Eligible women (1) were aged 30 to 64 years; (2) had not had a hysterectomy; (3) had a KPWA primary care clinician; (4) had been continuously enrolled for 3 years and 5 months or longer; and (5) had not had a Papanicolaou test within 3 years and 5 months. (Although screening guidelines recommend extending the routine screening interval from 3 to 5 years with Papanicolaou and HPV cotesting,^21^ none of the women would have been on a 5-year screening interval because cotesting was rarely performed at KPWA prior to August 2013.) Women who previously indicated they did not want to be contacted for research studies, had a pregnancy-related procedure or diagnosis code within 3 months or less, or had an “interpreter needed” flag (kit materials were in English) in the EMR were excluded.

Participants were randomly allocated 1:1 to the intervention or control group between February 25, 2014, and August 29, 2016. To meet our sample size goals during the study timeline, 1 year after randomization, control group participants were reassessed for eligibility and rerandomization.

Women in the control group received usual care outreach protocols to attend Papanicolaou screening. Outreach includes the birthday letter reminder, clinician-targeted automatic alerts for overdue women that persist until a Papanicolaou test is ordered or the alert is overridden, and primary care team communication (varies across clinics). Women randomized to usual care did not receive the mailed HPV kit or contact from the study team.

Women in the intervention group received usual care plus a mailed HPV self-sampling kit with a prepaid return envelope addressed to the KPWA clinical laboratory. Mailings included an invitation letter, research information sheet, and educational materials on how to self-collect and return a sample. Because HPV self-screening is not standard of care in the United States, the letter advised women to receive routine Papanicolaou testing regardless of whether they chose to complete HPV self-sampling. Women were informed that participation was voluntary and provided with a telephone number to call with questions or to opt out of having their individual-level medical record data used for research. To mirror KPWA’s prevention outreach protocols, if the kit was not returned within 3 weeks, study staff conducted up to 3 reminder calls. Samples were tested with the US Food and Drug Administration–approved Cobas 4800 HPV Test (Roche Diagnostics). Results were documented in the EMR and women’s primary care teams managed results communication and follow-up care. Women with negative or unsatisfactory results were advised to attend in-clinic Papanicolaou testing or cotesting. Women positive for high-risk HPV types other than HPV-16 or HPV-18 were advised to receive in-clinic cotesting. Women positive for HPV-16 or HPV-18 were recommended for immediate colposcopy, per the American Society for Colposcopy and Cervical Pathology management guidelines.^22^ Standardized protocols were developed by clinical and system-level collaborators to educate primary care teams on recommended follow-up (published previously^19^).

We used programmatic extraction and manual EMR review to identify 2 primary outcomes: histologically diagnosed CIN2+ and treated CIN2+. The 2 primary outcomes were selected based on clinical relevance. We did not adjust for multiple comparisons or have an order or gatekeeping strategy for testing given that these 2 outcomes are strongly correlated with each other (CIN2+ treatment cannot occur without a CIN2+ diagnosis), and it was highly unlikely that only 1 would be significant. To count as an outcome, CIN2+ had to be preceded by an abnormal screening result within 6 months after randomization or rerandomization. Diagnosed CIN2+ was captured within 6 months after an abnormal screening result, and treated CIN2+ was captured within 6 months after a CIN2+ diagnosis. With these windows, each woman was followed up for a maximum of 18 months after randomization.

Secondary outcomes included screening uptake and abnormal screening results, obtained from the EMR. Uptake was captured within 6 months after randomization or rerandomization and defined as (1) receiving a Papanicolaou screening or cotest; (2) self-sampling HPV-16 or HPV-18 positive (regardless of any subsequent in-clinic follow-up, because recommended management is to proceed directly to diagnostic evaluation^23^); (3) self-sampling HPV negative (regardless of any subsequent in-clinic follow-up, based on the expectation that programs implementing HPV self-sampling would not require in-clinic follow-up of negative results); or (4) self-sampling HPV positive for types other than HPV-16 or HPV-18 only or unsatisfactory and receiving a Papanicolaou screening or cotest. Abnormal screening results were captured within 6 months after randomization and defined as a result warranting repeated testing, surveillance, or immediate colposcopy before returning to routine screening. In addition to the prespecified secondary outcomes, we also measured screening initiation, defined as either receiving a Papanicolaou screening or cotest or self-sampling (without the requirement to follow up in-clinic after self-sampling HPV-positive for types other than HPV-16 or HPV-18 only or unsatisfactory).

Participant characteristics at randomization (age, race, ethnicity, health plan enrollment duration, time since last Papanicolaou test, census block median household income, travel time to primary care clinic, body mass index, tobacco use, and Charlson Comorbidity Index score^24^) were derived from EMR data. Outcome data were available for all participants, including women who did not return a kit or opted out of medical record review (details of data access and aggregation for these groups were published previously^19^).

The HOME trial was powered on the primary outcomes of diagnosed and treated CIN2+ assuming a 2-sided α of .05 and used assumptions based on the literature^25,26,27,28,29,30^ and 2012 preliminary data.^19^ We estimated we would identify approximately 17 600 eligible women over 2.5 years and would have 85% power to detect between-group differences in proportions with diagnosed CIN2+ (0.188% vs 0.036% [relative risk {RR}, 5.2] in the intervention and control groups, respectively) and 81% power for treated CIN2+ (0.170% vs 0.033% [RR, 5.2] in the intervention and control groups, respectively). We estimated 100% power to detect differences in abnormal screening results (1.09% vs 0.44% [RR, 2.5] in the intervention and control groups, respectively) and screening uptake (35.9% vs 8.8% [RR, 4.1] in the intervention and control groups, respectively).

### Statistical Analysis

We analyzed data using the intention-to-treat principle. Denominators for each group included all women randomized, minus the small number of women identified as ineligible after randomization (Figure 1). We described the distribution of participant characteristics by randomization group to identify any imbalances warranting adjustment in regression models. Outcome proportions in the intervention group were compared with the usual care group and RRs estimated using log-binomial regression. Absolute risk differences were estimated using binomial regression with an identity link. Robust variance estimates were used to account for within-participant correlation due to rerandomized participants contributing more than 1 observation period. Statistical significance was defined as a 2-sided *P* < .05.

As a prespecified exploratory analysis, positive predictive value of an abnormal screening test for detecting CIN2+ was estimated within each randomization group. Two different denominators were evaluated: women with abnormal screening results warranting colposcopic referral and women receiving colposcopy after an abnormal result.

In a post hoc analysis, we used Kaplan-Meier methods and log-rank tests to compare time from randomization to screening uptake in the control vs intervention group. Time to screening uptake was also compared among the control group vs intervention group women who returned HPV kits vs intervention group women with in-clinic screening only. Analyses were conducted using SAS statistical software version 9.4 (SAS Institute).

## Results

From February 25, 2014, to August 29, 2016, 16 590 women were randomized to the intervention group (n = 8283) or the control group (n = 8307) (Figure 1). From March 23, 2015, to August 29, 2016, 3231 women who were initially randomized to the control group and still eligible 1 year after randomization were rerandomized; between March 21, 2016, and August 29, 2016, 409 additional control group women were rerandomized a second time. Each rerandomization was treated as a distinct observation in the analyses. Therefore, the total number randomized was 20 230; 379 were retroactively excluded, and the remaining 19 851 (mean [SD] age, 50.1 [9.5] years) were included in the intention-to-treat analysis (9960 in the intervention group, 9891 in the control group). Baseline characteristics were similar between groups (Table 1).

**Table 1.** Baseline Characteristics of Intervention and Control Group Participants

Characteristic^a^	No. (%)	
	Intervention (n = 9843)^b^	Control (n = 9891)
Age at randomization, y		
30-34	808 (8.2)	794 (8.0)
35-39	932 (9.5)	915 (9.3)
40-44	1194 (12.1)	1185 (12.0)
45-49	1380 (14.0)	1374 (13.9)
50-54	1682 (17.1)	1707 (17.3)
55-59	1938 (19.7)	1943 (19.6)
60-64	1909 (19.4)	1973 (19.9)
Race		
White	7018 (76.4)	7111 (77.1)
Asian	893 (9.7)	880 (9.5)
Black or African American	438 (4.8)	431 (4.7)
Native Hawaiian or other Pacific Islander	151 (1.6)	139 (1.5)
American Indian/Alaska Native	147 (1.6)	145 (1.6)
>1 Race	285 (3.1)	283 (3.1)
Other	250 (2.7)	235 (2.5)
Unknown	661 (6.7)	667 (6.7)
Ethnicity		
Non-Hispanic	8710 (94.7)	8761 (94.8)
Hispanic	486 (5.3)	480 (5.2)
Unknown	647 (6.6)	650 (6.6)
Length of health plan enrollment, y		
3.4 to <5	2230 (22.7)	2240 (22.6)
5 to <10	3115 (31.6)	3045 (30.8)
≥10	4498 (45.7)	4606 (46.6)
Time since last Papanicolaou test (by length of enrollment), y		
Enrolled 3.4 to <5 y		
No.	2230	2240
No Papanicolaou test	1526 (68.4)	1530 (68.3)
>3.4 to <5	704 (31.6)	710 (31.7)
Enrolled 5 to <10 y		
No.	3115	3045
No Papanicolaou test	1056 (33.9)	1070 (35.1)
>3.4 to <5	1519 (48.8)	1468 (48.2)
5 to <10	540 (17.3)	507 (16.7)
Enrolled ≥10 y		
No.	4498	4606
No Papanicolaou test	694 (15.4)	666 (14.5)
>3.4 to <5	2186 (48.6)	2252 (48.9)
5 to <10	1143 (25.4)	1182 (25.7)
≥10	475 (10.6)	506 (11.0)
Women's US Census block, median household income, median (IQR), $	66 474 (50 343-85 000)	65 950 (50 536-83 949)
Travel time from women's home to primary care clinic, min		
<10	3254 (33.4)	3236 (33.1)
10 to <20	4086 (41.9)	4048 (41.4)
20 to <30	1407 (14.4)	1415 (14.5)
≥30	1004 (10.3)	1072 (11.0)
Unknown	92 (0.9)	120 (1.2)
Body mass index^c^		
<18.5	109 (1.3)	98 (1.2)
18.5-24.9	2238 (26.5)	2248 (26.7)
25-29.9	2168 (25.7)	2220 (26.3)
30-34.9	1549 (18.4)	1603 (19.0)
35-39.9	1119 (13.3)	1080 (12.8)
≥40	1248 (14.8)	1184 (14.0)
Unknown	1412 (14.3)	1458 (14.7)
Tobacco use		
Never	5237 (61.2)	5232 (61.3)
Current	1276 (14.9)	1290 (15.1)
Former	2041 (23.9)	2020 (23.6)
Unknown	1289 (13.1)	1349 (13.6)
Charlson Comorbidity Index score^24^		
0	7967 (80.9)	8052 (81.4)
1	1087 (11.0)	1128 (11.4)
2	432 (4.4)	385 (3.9)
≥3	357 (3.6)	326 (3.3)
Randomization year		
2014	4207 (42.7)	4254 (43.0)
2015	3570 (36.3)	3571 (36.1)
2016	2066 (21.0)	2066 (20.9)

Abbreviation: IQR, interquartile range.

^a^ Based on electronic medical record data.

^b^ Baseline characteristics are not available for 117 participants who opted out of electronic medical record review.

^c^ Calculated as weight in kilograms divided by height in meters squared.

### Primary Outcomes

**Figure 2.**
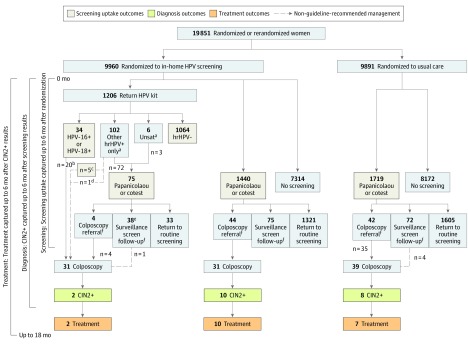
Diagram of Cervical Cancer Screening, Diagnosis, and Treatment Outcomes CIN2+ indicates positive results for cervical intraepithelial neoplasia grade 2 or higher; HPV, human papillomavirus; hrHPV, high-risk HPV; Unsat, unsatisfactory. ^a^Self-sampling other hrHPV+ only or unsatisfactory results required in-clinic follow-up to complete screening uptake. ^b^Of these 20 women, 15 went directly to colposcopy and 5 went to Papanicolaou test or cotest before colposcopy. ^c^These 5 women completed screening uptake by receiving colposcopy in the 6 month screening window. ^d^This 1 woman did not complete screening uptake because colposcopy was completed outside the 6-month screening window. ^e^Includes 2 women with home kit other hrHPV+ and Papanicolaou test with unknown result. Because unknown Papanicolaou test results were coded as normal for analysis, the final result is considered other hrHPV+ and Papanicolaou test negative. One of these women received a colposcopy. ^f^Follow-up recommendations were per national guidelines.^22^

Twelve patients with CIN2+ (0.12%) were detected in the intervention group (including 2 cases in women who returned a kit) vs 8 (0.08%) in the control group (Figure 2 and Table 2). The RR for CIN2+ detection was 1.49 (95% CI, 0.61-3.64) in the intervention vs control group. All women with CIN2+ (0.12%) were treated in the intervention group vs 7 in the control group (0.07%); the RR for CIN2+ treatment was 1.70 (95% CI, 0.67-4.32) in the intervention vs control group.

**Table 2.** Screening, Diagnosis, and Treatment Outcomes for the Intervention vs Control Group

Outcome	No. (%)		Relative Risk (95% CI)^a^	Absolute Risk Difference (95% CI)^a^
	Intervention Group (n = 9960)	Control Group (n = 9891)		
Primary outcome				
Cervical intraepithelial neoplasia grade 2 or higher	12 (0.12)	8 (0.08)	1.49 (0.61 to 3.64)	0.04 (−0.05 to 0.13)
Treatment received	12 (0.12)	7 (0.07)	1.70 (0.67 to 4.32)	0.05 (−0.04 to 0.14)
Secondary outcome				
Screening uptake^b^	2618 (26.3)	1719 (17.4)	1.51 (1.43 to 1.60)	8.9 (7.8 to 10.0)
Screening abnormal^c^	225 (2.3)	114 (1.2)	1.96 (1.57 to 2.45)	1.1 (0.8 to 1.5)

^a^ Robust variance estimates were used to account for within-participant correlation due to rerandomized participants contributing more than 1 observation period.

^b^ Screening uptake is defined as completion of screening episode; therefore, women who tested positive for human papillomavirus types other than 16 or 18 only or unsatisfactory on the mailed kit must have completed the additional step of in-clinic follow-up (Papanicolaou, cotest, or colposcopy).

^c^ Abnormal screening result defined as a result that warrants repeated testing, surveillance, or immediate colposcopy (per national guidelines^22^) before returning to a routine screening schedule.

### Prespecified Secondary Outcomes

Within the intervention group, 2618 women (26.3%) completed screening uptake vs 1719 (17.4%) in the control group. The RR for screening uptake was 1.51 (95% CI, 1.43-1.60) in the intervention vs the control group; absolute difference was 8.9% (95% CI, 7.8%-10.0%) (Figure 2 and Table 2). In the intervention group, 225 women (2.3%) received abnormal screening results vs 114 (1.2%) in the control group; abnormal screening RR was 1.96 (95% CI, 1.57-2.45) in the intervention vs control group.

Within the intervention group, 2646 women (26.6%) initiated screening; 1206 women (12.1%) initiated their screening episode by returning an HPV kit and 1440 women (14.5%) went directly to in-clinic screening (Figure 2). Among the 1206 women who returned a kit, 1098 attained screening uptake based on their HPV kit results alone, including 1064 (88.2%) with HPV-negative results and 34 (2.8%) with HPV-16– or HPV-18–positive results (Figure 2; eFigure in Supplement 2). The remaining 108 patients (9.0%) had kit results that were positive only for types other than HPV-16 or HPV-18 (8.5%) or unsatisfactory (0.5%) and required in-clinic follow-up to complete screening uptake; 80 of these 108 women (74.1%) attained screening uptake by attending in-clinic follow-up and 28 (25.9%) did not (Figure 2).

### Additional Analyses

Four women returned nonviable HPV kits, which were not tested (including 2 received beyond maximum specimen stability of 21 days after the collection date and 2 with visible stool in the collection tube). All 4 were notified and offered a replacement kit; 3 returned a second kit within 6 months of randomization, received HPV-negative results, and were counted in the screening uptake numerator. Additionally, 47 of the 1440 intervention group women (3.3%) who went directly to in-clinic screening subsequently returned an HPV kit; these HPV kit results did not affect primary or secondary outcomes.

**Figure 3.**
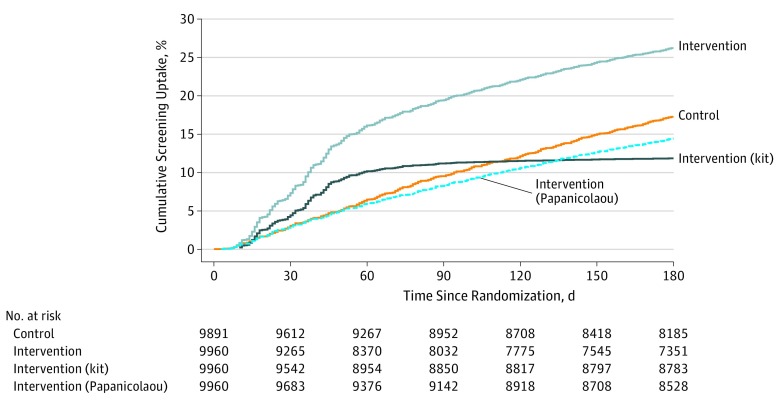
Cumulative Incidence of Screening Uptake by Allocation and Screening Modality Within the Intervention Group

Time to screening uptake was shorter in the intervention group vs the control group (log-rank *P* < .001) (Figure 3). Restricting to women who completed screening uptake, the median (interquartile range) number of days to screening uptake was 80 (43-129) for women in the control group and 46 (29-93) for women in the intervention group. Results for intervention group women who went directly to in-clinic screening (median [interquartile range], 78 [37-126] days) were similar to results for women in the control group, whereas time to screening uptake was shorter among women who returned an HPV kit (median [interquartile range], 37 [22-49] days). In all, 27.5% of kits were returned within 21 days (before reminder call initiation).

The number of abnormal screening results warranting colposcopic referral was similar in the intervention group (82 referrals, representing 36.4% of abnormal screening results) vs the control group (42 referrals, representing 36.8% of abnormal screening results) (Figure 2). Among those with results warranting colposcopy, 55 (67.1%) in the intervention group received colposcopy vs 35 (83.3%) in the control group. Within the intervention group, the proportion of abnormal results warranting colposcopy was similar in women who returned kits (38 of 106 [35.8%]) vs women who went directly to in-clinic screening (44 of 119 [37.0%]), and the number correctly receiving colposcopy was 24 of 38 (63.2%) in the kit returner group vs 31 of 44 (70.5%) in the direct to in-clinic screening group. Additional subgroup details, including colposcopies received against study recommendations or American Society for Colposcopy and Cervical Pathology management guidelines, are depicted in Figure 2.

Within the intervention group, the CIN2+ positive predictive value was 14.6% (95% CI, 7.0%-22.3%) (12 of 82) among women with abnormal screening results warranting colposcopy and 21.8% (95% CI, 10.9%-32.7%) (12 of 55) when restricting the denominator to women who received colposcopy. The positive predictive value for CIN2+ was 19.0% (95% CI, 7.2%-30.9%) (8 of 42) in women in the control group warranting colposcopy and 22.9% (95% CI, 9.0%-36.8%) (8 of 35) restricting to those who received colposcopy.

No unexpected and 5 expected^19^ (2 light bleeding; 3 discomfort) adverse events were self-reported.

## Discussion

In this randomized clinical trial, there were no significant differences in CIN2+ detection or treatment among underscreened women who received a mailed HPV self-sampling kit compared with usual care alone. However, mailing HPV kits significantly increased screening uptake and reduced time to screening compared with usual care.

We are unaware of other HPV self-sampling trials that evaluated treated CIN2+ cases as an outcome. However, many international trials have evaluated the effect of mailing HPV kits to underscreened women on CIN2+ detection, with mixed results.^15^ High-risk HPV–positive self-sampling triage strategies vary across settings and influence CIN2+ detection rates.^15^ Detection of CIN2+ was higher comparing HPV self-sampling with control groups in trials in which women were referred directly to colposcopy (pooled RR including 6 trials, 3.0; 95% CI, 1.9-4.8) compared with trials that triaged women to additional screening (usually cytology) (pooled RR including 7 trials, 1.8; 95% CI, 0.8-4.0).^15^ One Australian trial triaged HPV-16 and HPV-18–positive results directly to colposcopy and other high-risk-positive results to Papanicolaou testing (similar to this trial); 76% received appropriate follow-up after an HPV-positive self-sampling result, and no significant between-group differences in CIN2+ detection were reported (although the trial was not powered on this outcome).^15,31^ Although our trial was powered on CIN2+ detection and treatment outcomes, power calculations were based on assumptions of higher kit uptake (30%), lower control group screening uptake (9%), and higher follow-up adherence (particularly for HPV-positive kit results [89%]) than was observed.^19^ Additionally, HPV positivity (11.3%) was higher than estimated (7.5%), but comparable to that reported in the most recent meta-analysis (pooled positivity, 11%; 95% CI, 10%-12%).^15^

Among the intervention women who self-sampled, most with nonnegative results attended in-clinic follow-up (70%); however, only 59% with HPV-16 or HPV-18–positive results (highest risk for CIN2+) attended recommended colposcopy, and 74% with other high-risk HPV–positive or unsatisfactory results attended in-clinic follow-up. Our study protocol deployed several implementation strategies to encourage appropriate follow-up of abnormal results, including (1) specific guidance for clinicians in electronic test results, (2) monitoring communications between the clinician and patients of the recommended follow-up plan, and (3) sending electronic messages to clinicians when follow-up plans were not communicated. These strategies were also part of trial safety monitoring. Cervical cancer screening guidelines in the United States are complex and dynamic; for health care systems to successfully incorporate new screening modalities such as HPV self-sampling, additional resources to support screening, diagnosis, and treatment completion may be warranted.^32^

In a meta-analysis of 19 trials in countries with organized screening programs, 25% of underscreened women were screened (HPV kit or Papanicolaou test) after receiving an unsolicited HPV self-sampling kit in the mail,^15^ similar to the 26% uptake in the intervention group. However, in this trial, slightly more women who screened in the intervention group chose in-clinic screening (14%) over self-sampling (12%), whereas in the meta-analysis, more women who were mailed a kit chose self-sampling (19%) over in-clinic screening (6%).^15^ The absolute (9%) and relative (1.5) increases observed in screening uptake in the mailed HPV kit vs usual care alone group in our trial were within the lower range of estimates from international trials. The pooled absolute difference in the meta-analysis was 13% (95% CI, 10%-15%), and the pooled RR was 2.3 (95% CI, 1.9-2.9).^15^ Further investigation of US clinician and patient knowledge and attitudes are needed to understand why uptake of mailed HPV kit screening was lower than that observed internationally.

The HOME trial was strengthened by the pragmatic design, with direct integration of the intervention into existing clinical protocols. Data from EMRs were used to identify and randomize women who would be eligible for the intervention if it were adopted into clinical practice, thereby enhancing generalizability of the results. Results from the post hoc time-to-screen analysis add context to the findings; the rapid time to screening uptake associated with mailed kits is an additional benefit from a health system resource perspective.

### Limitations

This study has several limitations. A key difference between HOME and similar international trials was the need to consider the impact of the HPV self-sampling intervention on Healthcare Effectiveness Data and Information Set quality metrics.^2^ During the trial, only completed Papanicolaou tests counted toward the Healthcare Effectiveness Data and Information Set cervical cancer screening outcome. This directly affected the trial design in 2 important ways. First, to allow enough time for the delivery system to activate overdue women, it was necessary to delay mailing HPV kits for 5 months after the most recent annual preventive services reminder letter. Second, kit materials recommended all women attend Papanicolaou screening, even with HPV-negative self-sampling results, which may have negatively affected kit uptake. Furthermore, mailing kits as part of a research study may have had either a positive or negative effect on kit uptake. Results may have been different if (as in most international studies) HPV kits had been mailed to underscreened women as part of standard care and with the reassurance that negative results would not require in-clinic follow-up. An additional limitation was that kit materials were developed in English only, necessitating the exclusion of non–English-speaking women.

## Conclusions

Mailing HPV kits to underscreened women increased screening uptake compared with usual care alone, with no significant differences in clinical outcomes. Nearly three-quarters of women who received the intervention remained underscreened, and follow-up among self-testing HPV-positive women has room for improvement. Health care systems in the United States considering implementing primary HPV screening and outreach strategies with HPV self-sampling should focus on approaches to increase kit uptake and follow-up of positive results. Additional research efforts should consider a variety of strategies to increase cervical cancer screening.

## Notes

Supplement 1. **Trial Protocol**

Supplement 2. **eFigure.** Detailed Diagram of Cervical Cancer Screening Uptake and HPV Kit Results Within the Intervention Group

Supplement 3. **Data Sharing Statement**
