# Supplementary material for: Effect of Mailed Human Papillomavirus Test Kits vs Usual Care Reminders on Cervical Cancer Screening Uptake, Precancer Detection, and Treatment: A Randomized Clinical Trial
Source: JAMA Netw Open. 2019 Nov 6;2(11):e1914729. doi: 10.1001/jamanetworkopen.2019.14729 (PMC6865279; doi:10.1001/jamanetworkopen.2019.14729)
Supplement: Supplement 2. — eFigure. Detailed Diagram of Cervical Cancer Screening Uptake and HPV Kit Results Within the Intervention Group [file jamanetwopen-2-e1914729-s002.pdf]

Supplementary Online Content

Winer RL, Lin J, Tiro JA, et al. Effect of mailed human papillomavirus test kits vs usual care reminders on cervical cancer screening uptake, precancer detection, and treatment: a randomized clinical trial. *JAMA Netw Open*. 2019;2(11):e1914729. doi:10.1001/jamanetworkopen.2019.14729

eFigure. Detailed Diagram of Cervical Cancer Screening Uptake and HPV Kit Results Within the Intervention Group

This supplementary material has been provided by the authors to give readers additional information about their work.

© 2019 Winer RL et al. *JAMA Network Open.*

eFigure. Detailed Diagram of Cervical Cancer Screening Uptake and HPV Kit Results within the Intervention Group

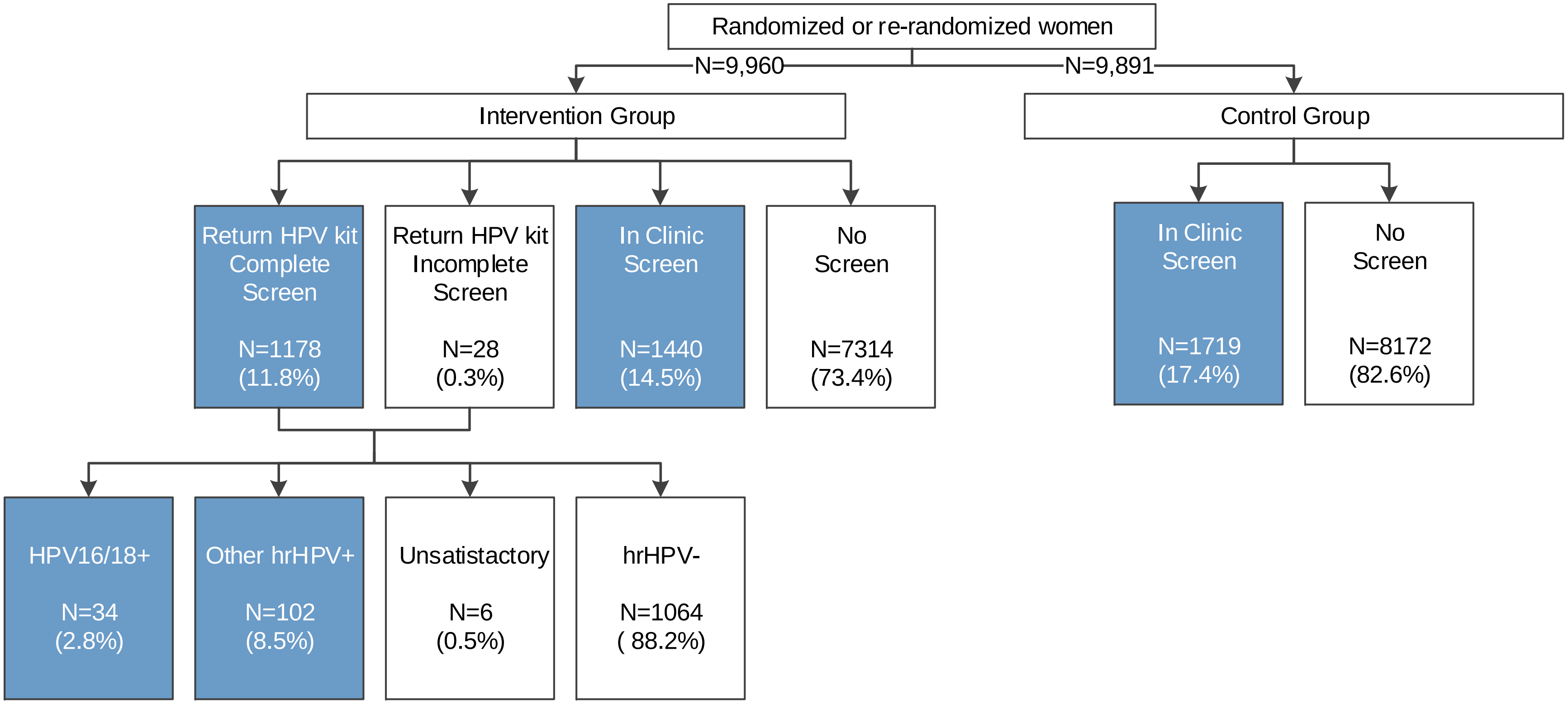

eFigure. Footnotes

Abbreviations: HPV, human papillomavirus; hrHPV, high risk human papillomavirus

© 2019 Winer RL et al. *JAMA Network Open.*
