# Supplementary material for: Effect of Mailed Human Papillomavirus Test Kits vs Usual Care Reminders on Cervical Cancer Screening Uptake, Precancer Detection, and Treatment: A Randomized Clinical Trial
Source: JAMA Netw Open. 2019 Nov 6;2(11):e1914729. doi: 10.1001/jamanetworkopen.2019.14729 (PMC6865279; doi:10.1001/jamanetworkopen.2019.14729)
Supplement: Supplement 3. — Data Sharing Statement [file jamanetwopen-2-e1914729-s003.pdf]

Data Sharing Statement

Winer RL, Lin J, Tiro JA, et al. Effect of mailed human papillomavirus test kits vs usual care reminders on cervical cancer screening uptake, precancer detection, and treatment: a randomized clinical trial. *JAMA Netw Open.* 2019;2(11):e1914729. doi:10.1001/jamanetworkopen.2019.14729

Data

Data available: Yes
Data types: Deidentified participant data, Data dictionary
How to access data: Requests for data must be sent to rlw@uw.edu.
When available: With publication

Supporting Documents

Document types: None

Additional Information

Who can access the data: Data will be made available to researchers whose proposed use of the data has been approved.
Types of analyses: Data will be made available for a specified purpose.
Mechanisms of data availability: Data will be made available without investigator support to researchers with adequate resources to cover the regulatory and data sharing costs. Data will be made available after approval of a concept proposal aligned with current data approvals, and with a signed data access agreement.
